# Comment on: A systematic review of the public's knowledge and beliefs about antibiotic resistance

**DOI:** 10.1093/jac/dkw141

**Published:** 2016-05-26

**Authors:** Dominique L. Monnet, Nabil Safrany, Nicole Heine, Charles Price

**Affiliations:** 1 Office of the Chief Scientist, European Centre for Disease Prevention and Control, Solna, Sweden; 2 Crisis Management and Preparedness in Health Unit, Directorate-General for Health and Food Safety, European Commission, Luxembourg, Luxembourg

Sir,

We read with interest the article by McCullough *et al*.^1^ presenting the results of their systematic review of quantitative and qualitative studies on the public's knowledge and beliefs about antibiotic resistance and antibiotics. The review included information from four major databases (MEDLINE, EMBASE, PsycINFO and CINAHL) without restrictions about study design or language, as well as forward and backward citation in Web of Science and Scopus. However, the review omitted two very large-size special Eurobarometer surveys performed by the European Commission in 2009 and 2013,^2,3^ which we think would be relevant to bring to your readers' attention.

Eurobarometer surveys aim to monitor the evolution of opinion of the public in the European Union (EU). Standard Eurobarometer surveys are performed two to five times a year for the European Commission by TNS Opinion & Social Network following a multi-stage random sampling design in each EU Member State.^4^ Special Eurobarometer surveys explore specific topics and are included as part of the polling waves for the standard Eurobarometer surveys.^4^ A special Eurobarometer survey on antimicrobial resistance was performed in November–December 2009 and included four questions on the knowledge of Europeans about antibiotics. In each EU Member State at least 1000 face-to-face interviews were performed (with the exception of Cyprus, Luxembourg and Malta, where at least 500 interviews were carried out in each country), making a total of ∼27 000 interviews. As part of its *Action Plan Against The Rising Threats From Antimicrobial Resistance*,^5^ the European Commission conducted a second special Eurobarometer survey on antimicrobial resistance in May–June 2013 using the same questions.

Overall, the percentage of Europeans that reported having taken an antibiotic orally during the 12 months preceding the survey decreased from 40% in 2009 to 35% in 2013.^3^

The results regarding public knowledge about antibiotics, overall for the EU as well as for each individual Member State, are compiled in Table 1. While differences in percentages observed for the EU overall between 2009 and 2013 are statistically significant due to the large sample size, differences observed for each individual Member State must be interpreted with caution, taking into account the indicative statistical margins provided in an annex to the report.^3^

**Table 1.** Public knowledge about antibiotics in 28 EU Member States, 2009 and 2013^2,3^

Country	No. of interviews		Percentage of correct answers for each proposal (increase or decrease in percentage points)							
			antibiotics kill viruses (correct answer: false)		antibiotics are effective against cold and flu (correct answer: false)		unnecessary use of antibiotics makes them become ineffective (correct answer: true)		taking antibiotics often has side effects such as diarrhoea (correct answer: true)	
	2009	2013	2009	2013	2009	2013	2009	2013	2009	2013
Austria	1001	1034	17	29 (+12)	26	33 (+7)	80	83 (+3)	67	72 (+5)
Belgium	1003	1006	56	52 (−4)	69	69 (=)	87	88 (+1)	66	67 (+1)
Bulgaria	1007	1025	22	21 (−1)	27	30 (+3)	81	78 (−3)	71	69 (−2)
Croatia	NA	1000	NA	48 (NA)	NA	55 (NA)	NA	87 (NA)	NA	66 (NA)
Cyprus	502	506	21	21 (=)	23	24 (+1)	94	92 (−2)	78	70 (−8)
Czech Republic	1096	1026	26	36 (+10)	53	63 (+10)	91	90 (−1)	57	63 (+6)
Denmark	1008	1010	52	57 (+5)	65	75 (+10)	96	97 (+1)	72	69 (−3)
Estonia	1000	1008	32	36 (+4)	48	54 (+6)	78	80 (+2)	78	77 (−1)
Finland	1041	1003	58	55 (−3)	72	74 (+2)	92	90 (−2)	82	75 (−7)
France	1005	1053	58	59 (+1)	64	64 (=)	87	92 (+5)	69	66 (−3)
Germany	1522	1505	31	36 (+5)	33	43 (+10)	84	87 (+3)	74	67 (−7)
Greece	1000	1002	24	25 (+1)	28	34 (+6)	92	93 (+1)	70	72 (+2)
Hungary	1017	1033	27	38 (+11)	29	37 (+8)	75	76 (+1)	50	62 (+12)
Ireland	1014	1001	45	51 (+6)	55	64 (+9)	89	85 (−4)	69	57 (−12)
Italy	1039	1025	29	33 (+4)	49	52 (+3)	65	68 (+3)	64	68 (+4)
Latvia	1004	1018	26	27 (+1)	35	38 (+3)	81	79 (−2)	68	65 (−3)
Lithuania	1027	1023	20	27 (+7)	29	38 (+9)	82	84 (+2)	74	69 (−5)
Luxembourg	502	502	46	58 (+12)	57	64 (+7)	85	89 (+4)	64	78 (+14)
Malta	500	500	18	23 (+5)	30	37 (+7)	94	90 (−4)	69	67 (−2)
Netherlands	1004	1013	52	56 (+4)	66	73 (+7)	93	94 (+1)	55	61 (+6)
Poland	1000	1000	33	32 (−1)	30	34 (+4)	85	85 (=)	74	78 (+4)
Portugal	1038	1007	14	19 (+5)	18	27 (+9)	84	79 (−5)	63	63 (=)
Romania	1008	1053	14	15 (+1)	28	33 (+5)	57	58 (+1)	50	45 (−5)
Slovakia	1047	1000	29	31 (+2)	46	53 (+7)	90	88 (−2)	74	75 (+1)
Slovenia	1017	1005	47	45 (−2)	63	61 (−2)	94	95 (+1)	73	74 (+1)
Spain	1023	1008	23	29 (+6)	32	44 (+12)	88	87 (−1)	70	61 (−9)
Sweden	1014	1000	73	74 (+1)	68	77 (+9)	97	98 (+1)	68	62 (−6)
UK	1322	1314	50	52 (+2)	65	70 (+5)	89	89 (=)	69	64 (−5)
EU-27^a^	26 761	27 680	36	40 (+4)	46	52 (+6)	83	84 (+1)	68	66 (−2)

NA, not available; =, no change.

^a^Excluding Croatia, which was not a member of the EU at the time these surveys were performed. Nevertheless, interviews were performed in Croatia in May–June 2013 and the results are reported in this table.

There was an increase between 2009 and 2013 in the proportion of people who responded that antibiotics do not kill viruses and are not effective against cold and flu. This is encouraging since the key messages of European Antibiotic Awareness Day, which was relayed by public awareness activities in EU Member States, mainly focused on these two issues.^6^ However, the Eurobarometer surveys indicated little change during the same period in the proportion of Europeans who answered correctly that unnecessary use of antibiotics is making them become ineffective or that taking antibiotics often has side effects such as diarrhoea.

The European Commission plans to perform further special Eurobarometer surveys in the future to assess changes in the knowledge and beliefs of Europeans about antibiotics. These will add to the growing knowledge base on this topic.^7,8^ We recommend that future reviews on public awareness about antimicrobial resistance should attempt to include relevant information and surveys that are published in the grey literature, such as those that may be carried out for the European Commission, the WHO and national governments.

## Transparency declarations

None to declare.

## References

[1] McCullough AR, Parekh S, Rathbone J et al A systematic review of the public's knowledge and beliefs about antibiotic resistance. *J Antimicrob Chemother* 2016; 71: 27–33. 26459555 10.1093/jac/dkv310

[2] European Commission. *Special Eurobarometer 338. Antimicrobial Resistance. November-December 2009*. Brussels: TNS Opinion & Social, 2010 http://ec.europa.eu/health/antimicrobial_resistance/docs/ebs_338_en.pdf.

[3] European Commission. *Special Eurobarometer 407. Antimicrobial Resistance. May-June 2013*. Brussels: TNS Opinion & Social, 2013 http://ec.europa.eu/health/antimicrobial_resistance/docs/ebs_407_en.pdf.

[4] European Commission. *Public Opinion*. http://ec.europa.eu/COMMFrontOffice/PublicOpinion/#p=1&instruments=STANDARD.

[5] European Commission. *Communication From The Commission To The European Parliament And The Council. Action Plan Against The Rising Threats From Antimicrobial Resistance. COM (2011) 748*. Brussels: European Commission, 2011 http://ec.europa.eu/dgs/health_food-safety/docs/communication_amr_2011_748_en.pdf.

[6] Earnshaw S, Mancarella G, Mendez A et al European Antibiotic Awareness Day: a five-year perspective of Europe-wide actions to promote prudent use of antibiotics. *Euro Surveill* 2014; 19: pii=20928. 10.2807/1560-7917.es2014.19.41.20928 25345519

[7] Wellcome Trust. *Exploring The Consumer Perspective On Antimicrobial Resistance*. London: Wellcome Trust, June 2015 http://www.wellcome.ac.uk/stellent/groups/corporatesite/@policy_communications/documents/web_document/wtp059551.pdf.

[8] WHO. *Antibiotic Resistance: Multi-Country Public Awareness Survey*. Geneva: WHO, 2015 http://apps.who.int/iris/bitstream/10665/194460/1/9789241509817_eng.pdf?ua=1.

